# Predictive Models of Maternal Harsh Parenting During COVID-19 in China, Italy, and Netherlands

**DOI:** 10.3389/fpsyt.2021.722453

**Published:** 2021-09-08

**Authors:** Madelon M. E. Riem, Paul Lodder, Jing Guo, Michelle Vrielink-Verpaalen, Marinus H. van IJzendoorn, Marian J. Bakermans-Kranenburg, Pietro De Carli

**Affiliations:** ^1^Behavioural Science Institute, Radboud University, Nijmegen, Netherlands; ^2^Clinical Child & Family Studies, Faculty of Behavioral and Movement Sciences, Vrije Universiteit, Amsterdam, Netherlands; ^3^Center of Research on Psychological & Somatic Disorders, Department of Medical and Clinical Psychology, Tilburg University, Tilburg, Netherlands; ^4^Department of Health Policy and Management, School of Public Health, Peking University, Beijing, China; ^5^Department of Psychology, Education and Child Studies, Erasmus University Rotterdam, Rotterdam, Netherlands; ^6^School of Psychology, Capital Normal University, Beijing, China; ^7^Department of Developmental and Social Psychology, University of Padua, Padua, Italy

**Keywords:** harsh parenting, COVID-19 pandemic, allomaternal support, father involvement, grandparents, cross-validation

## Abstract

**Background:** The COVID-19 pandemic drastically impacted on family life and may have caused parental distress, which in turn may result in an overreliance on less effective parenting practices.

**Objective:** The aim of the current study was to identify risk and protective factors associated with impaired parenting during the COVID-19 lockdown. Key factors predicting maternal harsh discipline were examined in China, Italy, and the Netherlands, using a cross-validation approach, with a particular focus on the role of allomaternal support from father and grandparents as a protective factor in predicting maternal harshness.

**Methods:** The sample consisted of 900 Dutch, 641 Italian, and 922 Chinese mothers (age M = 36.74, SD = 5.58) who completed an online questionnaire during the lockdown.

**Results:** Although marital conflict and psychopathology were shared risk factors predicting maternal harsh parenting in each of the three countries, cross-validation identified a unique risk factor model for each country. In the Netherlands and China, but not in Italy, work-related stressors were considered risk factors. In China, support from father and grandparents for mothers with a young child were protective factors.

**Conclusions:** Our results indicate that the constellation of factors predicting maternal harshness during COVID-19 is not identical across countries, possibly due to cultural variations in support from fathers and grandparents. This information will be valuable for the identification of at-risk families during pandemics. Our findings show that shared childrearing can buffer against risks for harsh parenting during COVID-19. Hence, adopting approaches to build a pandemic-proof community of care may help at-risk parents during future pandemics.

## Introduction

The COVID-19 pandemic drastically impacted on family life. Parents worried about their own and their families' health, job losses, and salary reductions, while keeping up their family life in social isolation. Moreover, because of (partial) school closures, families were suddenly faced with additional pressure of homeschooling their children. There may be considerable variability in how families deal with pandemic challenges and the extent to which they were impacted by COVID-19. For some families, the sequelae of the pandemic may lead to heightened psychological distress and, in turn, an overreliance on less effective parenting practices such as a harsh disciplinary style or even child abuse or neglect (1), with negative impact upon children's wellbeing. Other families, however, may manage relatively well. The current study therefore aims to identify risk and protective factors associated with impaired parenting during the lockdown amidst COVID-19. More specifically, we examined key family factors predicting maternal harsh discipline across three countries, China, Italy, and the Netherlands, using a cross validation modeling approach (2, 3). We particularly focused on the role of support from father and grandparents as a protective factor facilitating mothers' adaptability and buffering the effects of pandemic-related distress on caregiving behaviors. Harsh discipline, characterized by parental attempts to control a child using verbal violence (e.g., screaming) or physical punishment (e.g., hitting) (4), can be considered child emotional or physical maltreatment (5, 6). Given the long-term negative consequences of maltreatment for children's development (7) examining the predictive performance of factors contributing to harsh parenting is essential for identifying at-risk families and preventing detrimental effects on children during future pandemics.

### Kinship Networks and Harsh Parenting

The traditional African proverb “It takes a village to raise a child” may express an underlying truth (8). Mothers, or fathers, do not rear children on their own, but childrearing is usually embedded in larger kinship networks (e.g., grandparents, relatives, neighbors) and communities (schools, daycare centers) that offer support with childcare and/or education. This shared child care appears crucial for parental well-being and optimal child development. For example, involvement of nonresidential grandparents decreases parental stress and promotes children's well-being by stimulating prosocial behaviors and academic engagement (9). Similarly, support from relatives, friends, or neighbors reduces parental stress and lowers risk for child abuse and neglect (10). However, during COVID-19, support outside the family unit has abruptly been lost due to social distancing, closures of schools and daycare centers, and other pandemic and lockdown restrictions. Parents suddenly needed to rely solely on each other, yet distress triggered by the pandemic may interfere with the ability to provide adequate partner support (11). These circumstances may increase risk for harsh parenting practices.

### Pre-existing Vulnerabilities and Harsh Parenting

Families with pre-existing vulnerabilities may be particularly at risk for inadequate or harsh parenting during the pandemic. For example, economic hardship is an important factor contributing to risk for child abuse and neglect (6), but the level of risk that pandemic-related financial insecurities poses for parenting abilities likely depends on families' financial situation prior to the pandemic (11). Similarly, psychological distress induced by the pandemic may be particularly difficult to regulate for parents with pre-existing mental health problems, another well-known factor elevating risk for harsh parenting (6). Further, major life stressors, such as the COVID-19 pandemic, may lead to marital conflicts and dissolution or intimate partner violence (IPV) (11). The first studies on family functioning during COVID-19 report increased rates of IPV (12), which may spillover to and harm the child because violence is modeled as a way to deal with conflicts that may also emerge in the parent–child relationship (6). Lastly, environmental factors, such as overcrowded living conditions and lack of access to private outdoor space, may further elevate risk for abuse (13), in particular during lockdown amidst COVID-19 when families are required to stay home.

### Protective Factors and Harsh Parenting

Protective factors may, however, buffer the negative effects of COVID-19 on parenting abilities. These protective factors may either lie at the level of the individual parent, such as good (pre-existing) mental and physical health, or may be located in the family composition. One potentially important factor buffering the impact of crises, such as COVID-19, on maternal caregiving is allomaternal care, that is, childcare by adults other than the biological mother including fathers, grandparents, and other group members. Evidence from studies with high-risk families underscores how much allomaternal support matters. For example, father support reduces the adverse long-term effects of maternal depression during a child's infancy on later child behavior problem (14), suggesting that father involvement may compensate for maternal stress. In contrast, in families where father involvement is low or father is absent, as in the case of single mothers, mothers are at increased risk for abusing or neglecting their children (15, 16). Other family members may also offer allomaternal assistance, such as older siblings (17) and grandmothers (18). Research shows that the presence of a grandmother in the same household with a teenage mother increases the quality of mothering and, in turn, chances of a secure mother-infant attachment relationships (19). Similarly, having a grandmother at hand predicts improved health and cognition among low birth-weight infants (20), although under adverse conditions, such as extreme poverty, presence of grandparents may reduce life expectancy of offspring because they use scarce resources (21). These findings are in line with the grandmother hypothesis (22), stating that extended human female postmenopausal lifespan is an evolutionary adaptation that allows grandmothers to provide allomaternal care to their grandchildren in order to increase their fitness. Based on the grandmother hypothesis, it could be expected that shared childrearing may function as a resilience buffer in times of adversity and may also exert protective effects on mothers' caregiving abilities in the times of pandemics.

### Cultural Differences Across the Netherlands, Italy, and China

Although the cooperative nature of human childrearing is universal (23), it is influenced by cultural and economic factors (24). For instance, Western-European families are often only partly supported in child care by grandparents, but for example in low and middle-income countries grandparental involvement is much stronger (25). Moreover, the probability of grandparental co-residence with children and grandchildren is higher in non-western societies with traditions of filial piety (26). In China, co-residence with extended family, including grandparents, is common practice (27) and grandparents are often involved in full-time child care. In particular the grandmother forms an important child care provider for Chinese mothers who need to balance the competing demands of childcare and (full-time) work in the absence of adequate child care provisions (28). Chinese fathers also share care with mothers and are more likely than in the past to emotionally invest in their children because the single-child policy has weakened gender roles (29, 30). In contemporary China, child rearing is therefore considered a joint mission of mothers, fathers, and grandparents who together form an intergenerational parenting coalition (27).

During COVID-19, this extended family may be a source of resilience as the unexpected burden of the pandemic is shared among more people. Indeed, in a previous study with the same sample, we found that support from grandparents during the lockdown was associated with less maternal mental health symptoms (31). From an evolutionary perspective, it has been argued that human childcare practices in the context of extended families enhances children's survival by sharing the costs and load of raising children (18). Exclusive maternal care has even been considered out of step with nature (18) because, according to calculations of evolutionary anthologists, human children consume more than 13 million calories until they reach adulthood (32), which is far more than a mother can provide. Contrasting with extended families in China, in most western societies, including Italy and the Netherlands, the nuclear family is the traditional family, consisting of parents and children, living apart from grandparents and other relatives, e.g., (33). This may be disadvantageous during the lockdown. Non-residential grandparents, among those most vulnerable to COVID-19, were kept at distance from children and grandchildren, which increased their chances of survival but posed a problem for working parents who had grandparental childcare support prior to the pandemic.

For mothers in nuclear families, father involvement in childcare may be an important resilience factor buffering the effects of the pandemic on maternal caregiving. Yet, father involvement varies across cultures and paternal behaviors should not be presumed to have similar influences on mothers' caregiving behaviors across different cultural groups. For example, Craig and Mullan (34) showed that mothers' and fathers' work arrangements only predicted equal distribution of childcare between parents in countries supporting equal gender divisions. In Italy, where gender inequality is high and the rate of female employment is amongst the lowest in Europe (35), fathers do not re-adjust for mothers' working hours (34). Italian fathers tend to stick to unequal shares of childcare, promoting Italian families to rely on additional sources of allomaternal support. Due to modestly available formal child care and a ubiquitous feeling of compliance, it is customary that Italian grandparents assist parents and take care of their grandchildren on a regular basis (36).

Contrasting with Italy, the Netherlands shows a lower prevalence of the male breadwinner family. Dutch mothers often switch to a part-time job while fathers keep working full-time after becoming parents (37). This is also known as the one-and-a-half earner household (38). Although Dutch women still bear the largest part of the burden of household chores and child care activities in daily life (38), levels of gender equality are considered quite high (39). The Dutch formal child care system is used by a large proportion of parents (38, 40). Nevertheless, many parents in the Netherlands prefer to combine formal child care with some kind of informal child care, the most prevalent form of the latter being non-residential grandparents taking care of their grandchildren (40). Co-residence with grandparents is, however, uncommon in the Netherlands and COVID-19 separated many Dutch children from their non-residential grandparents, thus lowering sources of allomaternal support.

In addition to cultural differences in family composition, culture may also shape parenting practices since cultural values and norms may affect attitudes about raising children, which may in turn influence parent-child interaction (41). It is therefore important to take into account the role cultural context (42), when examining parenting during the COVID-19 lockdown. More specifically, parents may acquire certain beliefs on disciplinary styles, such as corporal punishment, within a cultural context and harsh discipline may occur more often in cultures or countries where practice of violence is viewed acceptable or normative. For example, a cross-cultural study on parenting across six countries Lansford, Chang (43) showed that harsh parenting is most prevalent in countries where physical discipline is perceived normative by parents. However, other research shows that there are far more cultural similarities than differences in parenting practices and that differences among cultural groups disappear when socioeconomic status is controlled (44).

### Aims and Hypothesis

In the current study we examined risk and protective factors predicting harsh parenting among mothers with children aged 1–10 years during the COVID-19 lockdown in China, Italy, and the Netherlands. Examining harsh parenting during the lockdown is important because expressions of violence in a family context has negative effects on children's development and psychosocial adjustment (45, 46). Our study extends a previous study in which we examined maternal mental health during the lockdown, but did not examine harsh parenting (31). Initial findings of research on the impact of COVID-19 point to increases in harsh parenting, with pandemic-related distress as a mediator (47). However, social and cultural context may either accentuate or minimize the impact of individual-level and family-level factors predicting harsh parenting. Hence, the constellation of parent and family characteristics as predictors of maternal harshness may not be replicable across countries. In the current study, maternal harsh parenting will therefore be examined across cultures by applying a cross-validation approach (2) for selecting models predicting maternal harshness in each country. Cross-validation allows accurate estimation of how a model would perform on other samples (3). In a predictive modeling context, cross-validation does not select the model predictors based on statistical significance, but based on their predictive performance. Predictive performance is especially important for the purpose of the current study, because in case of future pandemics involving lockdowns, identifying families at risk of harsh parenting or even child abuse is essential.

It can be expected that previously identified antecedents of child abuse and neglect, such as parental psychopathology, marital conflict, low socioeconomic status, low father involvement, a large number of children, and poor housing (6, 15, 16, 48), also enhance risk for harsh caregiving in the time of COVID-19. However, in addition to these previously identified antecedents, risk factors more closely related to acute COVID-19-related stress, such as COVID-19 related concerns about health and work increase, may further elevate risk for maternal harshness, whereas allomaternal support may exert protective effects on mothers' caregiving abilities. Hence, our first hypothesis was that previously identified risk factors for child abuse and COVID-19 related stress about health and work would increase risk for harsh maternal caregiving, whereas involvement of father and (co-residential) grandparents would buffer against risk. Second, we hypothesized, in line with the grandmother hypothesis (22, 49, 50), that grandparental involvement would be particularly beneficial for mothers with young children who are still highly dependent on the physical and emotional availability of caregivers. Thirdly, we expected that high levels of allomaternal support, i.e., support from both fathers and grandparents, facilitate mothers' adaptability and mitigate the effects of pandemic-related distress on caregiving. Lastly, we hypothesize that mothers in the three countries may be differently impacted by the pandemic. This expecation was also based on our previous finding that grandparental support during the lockdown lowers risk for mental health symptoms for Chinese mothers, but not for Italian and Dutch mothers (31). Although child physical abuse is a global phenomenon, unaffected by cultural–geographical factors (51), factors predicting harsh parenting during COVID-19 may differ across countries due to cultural variations in allomaternal support. Thus, we tested the hypothesis that the constellation of factors contributing to maternal harsh parenting during COVID-19 is subject to influences of family composition and may therefore vary across countries.

## Methods

### Participants and Design

Dutch, Chinese, and Italian parents aged 18 years or older with children between 1 and 10 years were invited to participate by completing an online survey. In each country, parents were recruited by contacting elementary schools. In the Netherlands and Italy, parents were also recruited by contacting day care centers using social media advertisements (facebook, linkedin, twitter). Dutch parents were also recruited by distributing the questionnaire among parents who were members of the Dutch I&O research panel (www.ioresearch.nl). The minimum sample size was 400 parents in each country, providing sufficient power to detect moderately sized correlation coefficients (power = 0.80, *r* = 0.20) between harsh parenting and each of the predictor variables, but we strived for larger sample sizes. Parents who completed the questionnaire but did not meet the inclusion criteria (e.g., they had only children older than 10 years, *N* = 8 Dutch parents, *N* = 47 Chinese parents) were excluded. The final sample consisted of 1,156 Dutch parents, 674 Italian parents, and 1,243 Chinese parents. Fathers were excluded from the analyses for the purpose of the current study, resulting in a sample of 900 Dutch, 641 Italian, and 922 Chinese mothers for this study. Characteristics of the Dutch, Chinese, and Italian samples are presented in Table 1. Permission for the study was obtained from the local ethics committees of the School of Social and Behavioral Sciences of Tilburg University, Department of Psychology of Padua University, and Peking University Medical Ethics Board. Participants gave informed consent and were given a chance at winning a gift voucher.

**Table 1:** Characteristics of Chinese, Italian, and Dutch mothers/families during the COVID-19 pandemic.

**Characteristic**	**IT**	**NL**	**CH**	***p*** **-value**	***Eta^2^***	***Cramer V***
	**(N=641)**	**(N=900)**	**(N=922)**			
Age mother	38.1 (5.56)	37.2 (5.18)	35.3 (5.67)	<0.001	0.04	
Marital status				<0.001		0.09
Single	9 (1.4%)	38 (4.2%)	4 (0.4%)			
Living together/Married	612 (95.5%)	828 (92.0%)	876 (95.0%)			
Divorced	13 (2.0%)	17 (1.9%)	26 (2.8%)			
Widow	1 (0.2%)	2 (0.2%)	4 (0.4%)			
Other	6 (0.9%)	15 (1.7%)	12 (1.3%)			
Education				<0.001		0.32
Primary school	0 (0%)	5 (0.6%)	18 (2.0%)			
Secondary school	211 (32.9%)	59 (6.6%)	242 (26.2%)			
College	128 (20.0%)	609 (67.7%)	516 (56.0%)			
University	275 (42.9%)	180 (20.0%)	115 (12.5%)			
Postgraduate	27 (4.2%)	47 (5.2%)	31 (3.4%)			
Employment				<0.001		0.22
Employed	474 (73.9%)	645 (71.7%)	863 (93.6%)			
Unemployed	104 (16.2%)	146 (16.2%)	57 (6.2%)			
Student	10 (1.6%)	22 (2.4%)	2 (0.2%)			
Other/unknown	53 (8.3%)	87 (9.7%)	0 (0%)			
Household income (Euros)				<0.001		0.34
<10.000	25 (3.9%)	13 (1.4%)	129 (14.0%)			
10.000–20.000	67 (10.5%)	49 (5.4%)	171 (18.5%)			
20.000–30.000	134 (20.9%)	57 (6.3%)	154 (16.7%)			
30.000-60.000	220 (34.3%)	315 (35.0%)	226 (24.5%)			
60.000–160.000	64 (10.0%)	309 (34.3%)	197 (21.4%)			
160.000–250.000	0 (0%)	20 (2.2%)	27 (2.9%)			
>250,000	1 (0.2%)	4 (0.4%)	18 (2.0%)			
Unknown	130 (20.3%)	133 (14.8%)	0 (0%)			
House with garden	404 (63.0%)	882 (98.0%)	506 (54.9%)	<0.001		0.44
Marital conflict Median [min, max]	2.00 [1.0, 6.0]	1.50 [1.0, 6.0]	2.00 [1.0, 6.0]	<0.001	0.02	
Number of Children				<0.001		0.19
1	261 (40.7%)	232 (25.8%)	409 (44.4%)			
2	308 (48.0%)	440 (48.9%)	450 (48.8%)			
3	60 (9.4%)	170 (18.9%)	59 (6.4%)			
4	9 (1.4%)	41 (4.6%)	4 (0.4%)			
5	1 (0.2%)	10 (1.1%)	0 (0%)			
6	2 (0.3%)	7 (0.8%)	0 (0%)			
Age youngest child Median [Min, Max]	3 [0, 10]	4 [0, 10]	6 [0, 10]	<0.001	0.06	
Childcare during COVID-19: grandparents	117 (18.3%)	85 (9.4%)	494 (53.6%)	<0.001		0.44
Childcare during COVID-19: other parties	38 (5.9%)	127 (15.1%)	30 (3.3%)	<0.001		0.18
Childcare before COVID-19: other parties	401 (62.6%)	598 (66.4%)	173 (18.8%)	<0.001		0.45
Father involvement	2.23 (0.554)	2.30 (0.620)	2.51 (0.673)	<0.001	0.07	
Harsh discipline median [Min, Max]	5 [0, 32]	2 [0, 32]	4 [0, 40]	<0.001	0.04	
General psychopathology median [Min, Max]	1.89 [1, 4.59]	1.36 [1, 4.74]	1.11 [1, 5]	<0.001	0.28	
Work related changes	3.91 (2.19)	2.74 (1.66)	4.91 (3.66)	<0.001	0.11	
Work related stress	6.90 (2.63)	4.58 (2.83)	4.75 (3.09)			
COVID-19 health concerns	5.44 (2.93)	4.11 (2.48)	5.27 (3.19)	<0.001	0.04	
Mean daily COVID-19 deaths during data collection (WHO)	217	111	0			
Cumulative deaths last day data collection (WHO)	34,223	5,422	4,643			

### Procedure

Data was collected using Qualtrics in Italy and the Netherlands, and using a web-based platform (https://www.wjx.cn/app/survey.aspx) in China. Timeframes for data collection were: April 17–May 10 2020 for the Netherlands, April 21–June 13 2020 for Italy, and April 21–April 28 2020 for China. During these timeframes, governmental pandemic measures in the three countries included: remote working, keeping social distance from others, and schools and daycare centers were closed. In each country, in particular older people were advised to keep distance. Dutch people were allowed to leave their home if they had no COVID-19 diagnosis or symptoms and if they had not been exposed to infected others. Also in Italy people were gradually allowed to leave their home during the period of data collection (after May 4). The Chinese data was collected in the aftermath of the COVID-19 peak, but pandemic restrictions were comparable to the Netherlands and Italy. Similar to Italy and the Netherlands, people worked remotely, were allowed to leave their home, but were advised to keep social distance. We focused on recruitment in the regions that were most affected by COVID-19, that is, Northern Brabant (the Netherlands), Lombardy (Italy), and Henan, Hubei, and Shenzhen city (China), although parents from others regions in Italy and the Netherlands were also allowed to participate.

### Measurements

#### Parent-Child Conflict Tactics Scale

The Parent-Child Conflict Tactics Scale (CTSPC) (52) was administered in order to assess maternal harsh disciplinary style. The CTSPC measures psychological and physical maltreatment and neglect of children by parents, as well as sensitive modes of discipline. For the purpose of the current study, we focused on the subscales psychological aggression (five items) and physical assault subscales (four items). An example item of the psychological aggression scale is “I shouted, yelled, or screamed angrily at my child”, while an example item of the physical assault scale is “I slapped my child on the hand, arm, or leg”. One item of the original 5-item physical assault subscale was excluded in order to prevent feelings of discomfort in parents. Mothers rated how often they used the different types of disciplinary behavior in the past two weeks on a 6-point scale, ranging from never to ≥5 times). A harsh parenting score was calculated by summing the nine items of the psychological aggression and physical assault subscales. Confirmatory factor analyses for ordered categorical item scores indicated that a 1-factor harsh discipline model fitted the data (RMSEA (95% CI) = 0.067–0.08; CFI = 0.969; SRMR = 0.057). The estimated reliability was good (McDonald's Omega Ω = 0.99).

#### Allomaternal Support

Participants were asked to indicate whether or not they received support in child care from residential or non-residential grandparents. In Italy and the Netherlands, very few mothers reported receiving support from residential grandparents (Italy: 3.0%, *N* = 19, the Netherlands: 1.1%, *N* = 10) whereas approximately half of the Chinese sample reported a cohabitating grandparent (China: 53.1%, *N* = 490). Despite governmental recommendations to keep safe distance from grandparents, some mothers reported child care by nonresidential grandparents (Italy: 15.3%, *N* = 98, the Netherlands: 8.3%, *N* = 75, China: 0.5%, *N* = 4). Since the number of parents receiving support for nonresidential grandparents was very low, we decided to combine support for residential and nonresidential grandparents. In addition, involvement of father in household management/tasks and child care was assessed by asking the degree of maternal and paternal contributions to 20 household chores or child care activities. Activities included: homeschooling, clearing the table, large purchases, loading dishwasher/washing dishes, grocery shopping, cooking, small purchases, paying bills, cleaning up house, chores in and around the house, making beds, washing and dressing up child, cleaning the house, bringing child to bed, soothing child at night, making list for grocery shopping, washing clothes, ironing, washing car, taking out trash. Mothers were asked to rate their own contribution and the contribution of their child's father to these tasks in the past week on a scale ranging from 1 (almost exclusively mother) to 5 (almost exclusively father). Cronbach's Alpha was 0.90. Mean scores were calculated, with higher scores representing greater involvement of father. The average of these 20 item scores was used as a measure of father involvement.

#### Work Changes and Stress

Participants reported on changes in their employment that occurred due to the COVID-19 outbreak, such as loss of hours or job or decreased job insecurity. Mothers reported on the following work changes: moved to remote working, loss of hours, decreased pay, loss of job, decreased job security, disruptions due to childcare challenges, increased hours, increased responsibilities, increased monitoring and reporting, loss of health insurance, reduced ability to afford childcare, reduced ability to afford rent/mortgage, having to fire or furlough employees, decrease in value of retirement, investments, or savings. A total score was calculated by summing reported negative changes. In addition, participants reported on the level of distress they experienced due to the employment and financial impacts of the COVID-19 outbreak on a Likert scale ranging from 1 (no distress) to 10 (severe distress). The correlation between work changes and work-related distress was *r* = 0.35, *p* < 0.001.

#### General Psychopathology

Mental health was measured with the Brief Symptom Inventory 18 (BSI-18, omitting suicidality), measuring somatization (six items), depression (five items), and anxiety (six items), and a subset of 10 questions of the posttraumatic stress disorder (PTSD) checklist for DSM-5. Because these four latent mental health constructs were highly correlated (range r 0.776–0.961), aggregate psychopathology scores were computed by averaging all 27 item scores. Confirmatory factor analysis for ordered categorical data supported this decision by indicating that one general psychopathology factor adequately explained the correlational structure of the four latent psychopathology factors (RMSEA = 0.06; CFI = 0.974; SRMR = 0.043).

In addition, health concerns specifically related to COVID-19 were measured. Parents rated the level of distress they experienced due to COVID-19 related symptoms or potential exposure they had or their family or friends had. A score representing general COVID-19-related health concerns was calculated by averaging the two items measuring concerns for self and family and friends. The correlation between health concerns for self and health concerns for others was *r* = 0.825 *p* = <0.001.

### Statistical Analysis

All analyses were conducted using the freely available software R [version 4.0.2; (53)]. Means and standard deviations were computed for continuous and normally distributed characteristics, and median and range were used for non-normally distributed continuous variables. Categorical characteristics were expressed in frequencies and percentages. For continuous characteristics, the differences between the three countries were tested using one-way analyses of variance and interpreted using the Eta squared effect size. Chi-square tests were used for categorical characteristics and interpreted using Cramer's V effect size). The 9-item harsh discipline scale was used as the primary outcome measure in all cross validation analyses. The R-package xvalglms (2) allowed for conducting linear regression analyses using K-fold cross validation. Cross validation allows for estimating how a model would perform on other samples. This out-of-sample predictive performance is more accurately determined by cross validation than by traditional model fit measures such as R-squared (3). One advantage of cross-validation is that it more accurately tests out-of-sample predictive performance than by traditional model fit measures such as R-squared. Other advantages of cross validation are that (1) it prevents overfitting the model to the idiosyncrasies of the data collected, (2) often violated regression model assumptions [e.g. linear relation between a predictor and the outcome; homoscedastic and normally distributed residuals; (2)] are no longer required, and (3) it does not rely on *p*-values to determine the significance of a predictor, thereby preventing the problems related to p-hacking [e.g., inflated false positive rates; (54)]. Our cross validation analyses involved two steps. In the first step, ten folds and 200 repeats were used to determine which combination of the 15 predetermined effects showed the best predictive performance in each of the three countries. This project's open science framework page includes a list of the predetermined effects, as well as the R-scripts (https://osf.io/9w8td). The inclusion or exclusion of each of those 15 effects corresponds to a total of 2^15^ = 32,768 different regression models. Given that interaction effects were investigated, incorrectly specified models were excluded (i.e., those including interaction effects without the corresponding main effects), resulting in a final amount of 13,311 regression models. For each country, each of those 13,311 models was fit to each of the 200 repeatedly drawn training datasets. In each repeat, the full data was split randomly into ten parts. One of those parts served as the training data, the remaining nine as the test data used to validate the model estimated on the training data. The predictive performance on these test datasets was evaluated in terms of the root mean square error of prediction (RMSE_p_). For each country, the model that most often showed the lowest prediction error across the 200 repeats was considered to have the best predictive performance. In the second step of our analyses, the best fitting model of each of the three countries was validated on the data of the other two countries, in order to determine the cross-cultural validity of the factors predicting harsh discipline in each country. For each country's winning model, the importance of the predictors was evaluated based on standardized regression coefficients resulting from a robust regression analysis to handle the violation of the homoscedastic residuals assumption in standard OLS regression.

## Results

### Descriptive Characteristics

**Figure 1:**
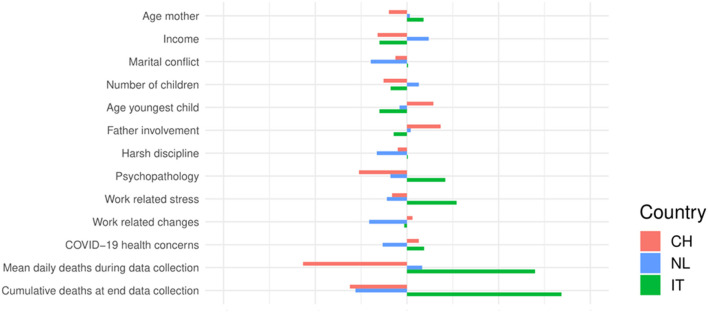
Differences between countries on continuous sample characteristics. Differences between countries were expressed as the deviation from the grand mean in terms of Z-scores.

**Figure 2:**
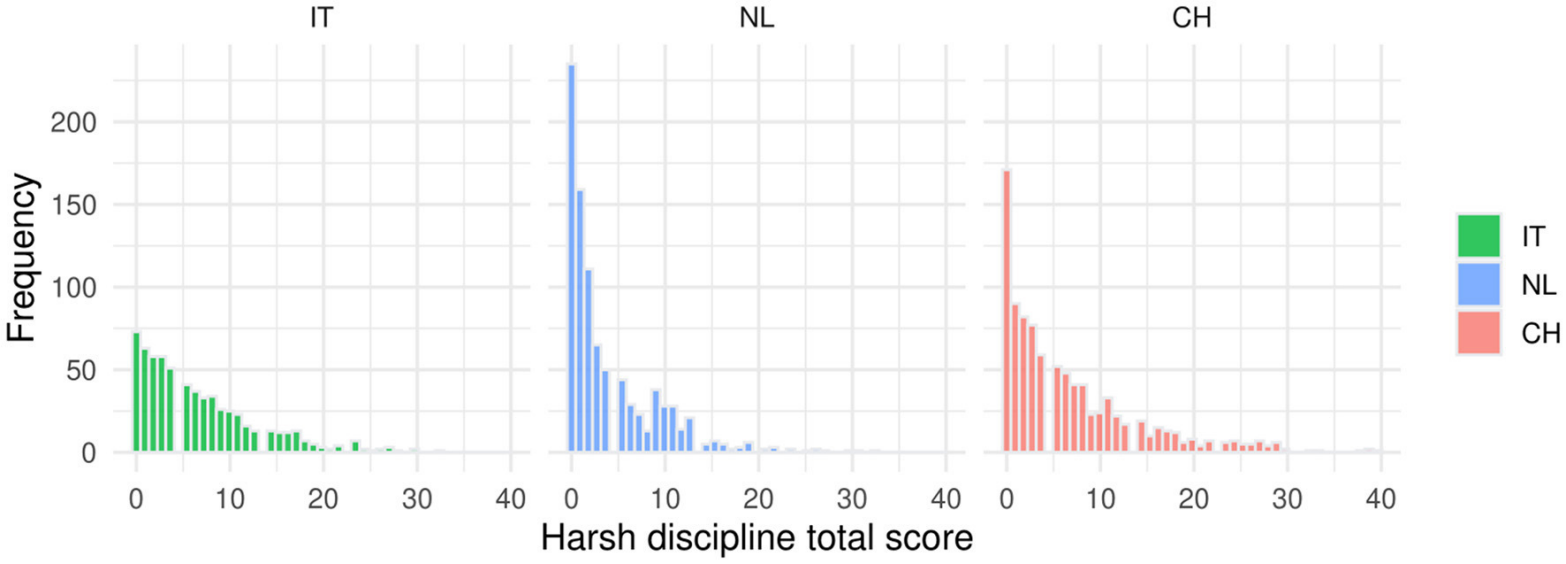
Harsh discipline total score distributions for Italian, Dutch and Chinese mothers.

Table 1 presents the characteristics of Chinese, Italian, and Dutch families during the COVID-19 pandemic, including age of the mother, marital status, and employment. Significant differences between countries were found for almost all characteristics, because the large sample size of the study makes these statistical tests sensitive to detect very small differences between countries. Effect sizes of between-country differences on socioeconomic/demographic variables (age youngest child, age mother, education, marital status, number of children, employment) were small. However, as expected, there were large differences between countries in childcare involvement of grandparents. In China, 53.6% of the mothers indicated that one or more grandparents provided support, whereas this percentage was considerably lower in both the Netherlands (9.4%) and Italy (18.3%). Figure 1 provides a visual representation of the differences between countries on the continuous characteristics listed in Table 1. See Supplementary Table 1 for additional information regarding quarantine situation and COVID-19 diagnoses among parents. Figure 2 shows for each country the distribution of the harsh discipline total scores. Harsh parenting differed significantly between the three countries: Dutch mothers used less harsh parenting than Chinese and Italian mothers. Supplementary Tables 3, 4 and 5 present the correlations between the two subscales of the CTSPC (psychological aggression and physical assault), childcare involvement of fathers, work-related distress, depression, anxiety, and posttraumatic stress disorder in the Dutch, Italian, and Chinese samples.

### Cross Validation

Table 2 shows for each country the top three regression models in terms of minimizing the prediction error (RMSE) in the cross validation analyses. The number of wins indicates the percentage of the 200 cross validation repeats a particular model showed the lowest prediction error (RMSE) of all 13,311 investigated models. The cross validation procedure identified a unique winning model for each of the three countries. In Italy, number of children, education, house with garden, general psychopathology, and marital conflict were important predictors. In the Netherlands, the following predictors were found: number of children, work change, general psychopathology, marital conflict. In China, income, education, work stress, general psychopathology, marital conflict, father involvement and the interaction between grandparental involvement and age youngest child were important predictors (see Supplementary Table 2).

**Table 2:** The standardized regression coefficients (β) and Wald test *p*-values according to robust regression analyses, including for each country only the predictors of the winning model.

**Predictor**	**Italy**		**Netherlands**		**China**	
	**β**	***p*** **-value**	**β**	***p*** **-value**	**β**	***p*** **-value**
Number of children	0.152	<0.001*	0.077	0.012*		
Education	−0.058	0.131			−0.067	0.041*
Income					−0.05	0.109
House with garden	0.073	0.059				
Work changes mother			0.119	<0.001*		
Work stress mother					0.137	<0.001*
General psychopathology	0.147	<0.001*	0.195	<0.001*	0.266	<0.001*
Marital conflict	0.236	<0.001*	0.072	0.028*	0.123	<0.001*
Father involvement					−0.118	<0.001*
Grandparents childcare					−0.035	0.262
Age youngest child					−0.058	0.066
Grandparents childcare *.					0.076	0.012*
Age youngest child						
Adjusted model *R*^2^	11.4%		7.1%		13.6%	

*Adjusted model R^2^ based on a linear ordinary least squared regression model*.

* *Wald test p < 0.05*.

**Figure 3:**
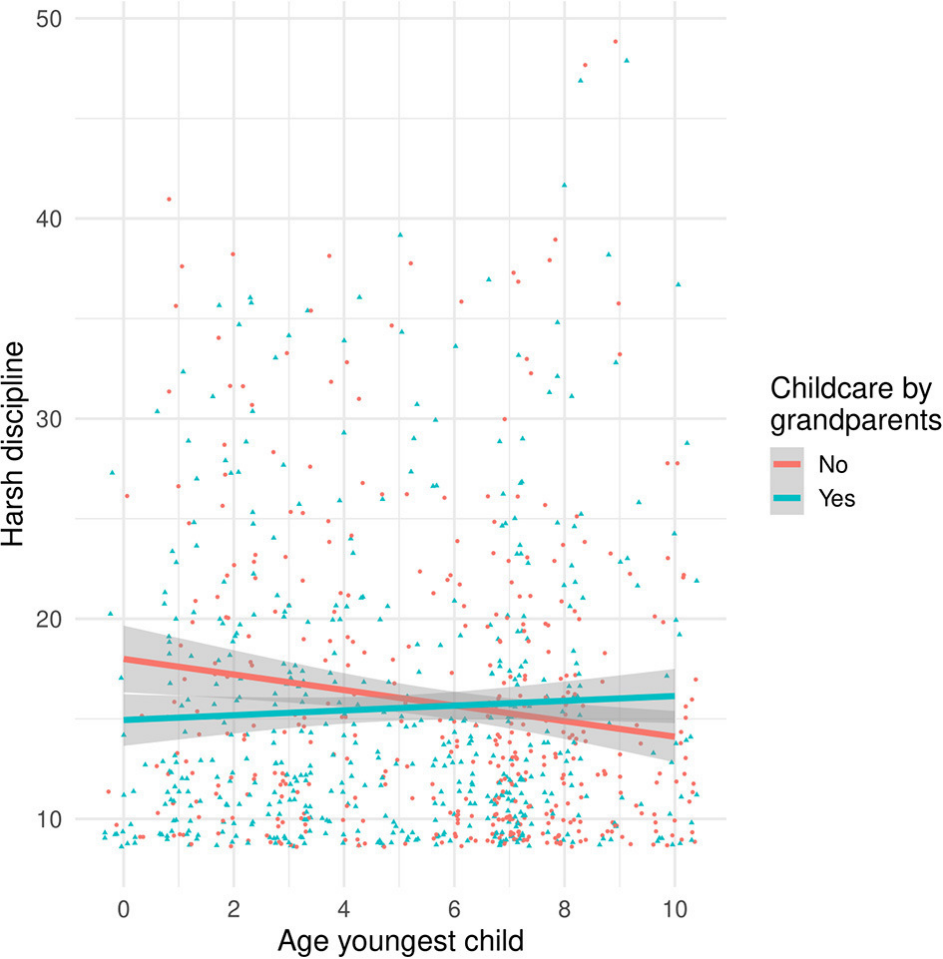
Scatterplot showing the Interaction between age of the youngest child and Chinese grandparental childcare (separate lines) on harsh discipline (y-axis).

Table 2 presents the standardized regression coefficients (β) and Wald test *p*-values according to three robust regression analyses, including for each country the predictors of the winning model identified through cross validation. In all countries, marital conflict and psychopathology showed a substantial positive association with harsh parenting, although there were considerable between-country differences in the identified predictors. In line with our expectations, harsh parenting was partly explained by the interaction between childcare offered by grandparents and age of the youngest child. Figure 3 illustrates this interaction effect, showing that grandparental childcare was associated with less harsh parenting by Chinese mothers, especially when the youngest children were still young.

**Figure 4:**
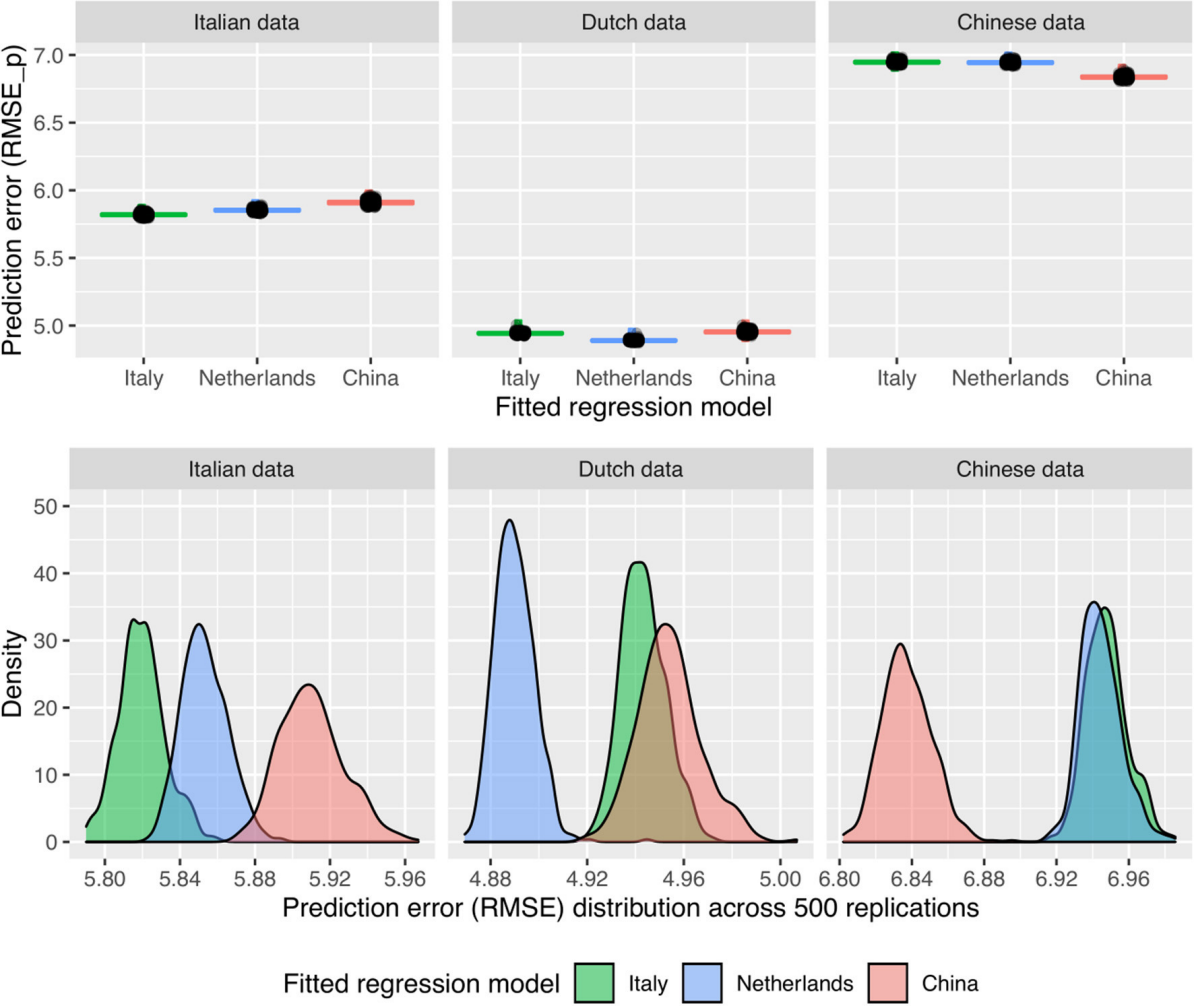
Boxplots (upper row) and density plots (bottom row) showing the distribution of RMSE when fitting each country's best model to the dataset of each country.

To determine the cross-cultural predictive validity of each country's winning model, a second series of cross validation analyses were conducted, evaluating the predictive performance of each winning model when predicting harsh parenting in the other two countries. Figure 4 visualizes the resulting prediction error distributions for each of the fitted top models and each of the three datasets. Unsurprisingly, for each dataset, the country's own best model showed the lowest prediction error in 100% of the cross validation repeats. The distributions in the bottom row of Figure 4 show that the Dutch and Italian models perform poorly in predicting harsh parenting in China. Interestingly, the overlapping distributions of the Dutch and Italian models in the Italian data suggests that the Dutch predictors can reasonably well predict harsh care of Italian mothers.

## Discussion

In the current study we examined risk and protective factors predicting maternal harsh parenting during the COVID-19 lockdown in China, Italy, and the Netherlands. We applied a cross-validation approach (2) for selecting which combination of 15 predetermined effects showed the best predictive performance in each country. Predictive modeling pointed to marital conflict and maternal psychopathology as shared risk factors predicting harsh parenting in each of the three countries. Despite these common factors, cross-validation identified a unique winning model for each of the three countries, thus indicating that the winning models with the best predictive performance differed between countries. In the Netherlands, work changes and number of children in the home predicted harsh parenting in addition to psychopathology and marital conflict, whereas in Italy, number of children, education, and house with garden were considered important predictors of maternal harsh parenting. In contrast, harsh parenting used by Chinese mothers was best predicted by education, income, and work-related stress of the mother. In addition, father involvement and grandparental involvement for mothers with a young child were considered important protective factors lowering risk for harsh parenting in China. Our findings extend our previous study in which we examined maternal mental health during the lockdown in China, Italy, and the Netherlands, but did not assess harsh parenting (31). Results indicate that, in addition to marital conflict and maternal psychopathology as shared risk factors, models predicting harsh parenting during COVID-19 include distinct risk factors that are not replicated across cultures, possibly due to cultural variations in family composition and allomaternal support. Hence, although harsh parenting is a global phenomenon (51), the constellation of factors predicting maternal harshness during COVID-19 is not identical.

First results of COVID-19 studies indicate that the pandemic drastically impacted on family life and that COVID-19 related distress can increases harsh parenting practices [e.g., (47)]. Our cross-validation results extend results of initial studies by indicating that there were considerable between-country differences in the identified predictors of maternal harshness. In our cross-validation approach, model predictors were not selected based on statistical significance, but based on their performance in predicting harsh parenting in each country. This predictive modeling context contrasts with the traditional explanatory data analysis approach used by previous COVID-19 studies and enables the identification of a risk factor model that most accurately predicts harsh care during the lockdown in each of the three countries. Our finding that each country has a unique constellation of factors predicting harsh parenting indicates that we should be careful with generalizing findings on disrupted parenting during the lockdown to other countries. The predictive performance of models predicting harsh care during COVID-19 is not the same across countries, implying that there is no universal risk factor model that can be used for the identification of at-risk families across countries.

In line with our expectations, we found that grandparental involvement lowered the risk for harsh parenting among Chinese mothers. Interestingly, grandparent involvement interacted with age of the child. The grandparent effect was particularly pronounced for Chinese mothers with younger children, which is in line with previous studies showing that grandparental involvement is particularly advantageous for children in the post weaning phase. For example, (50) showed a positive grandmother effect on the nutritional status of Aka children in Congo, with their effect most evident during the critical 9–36 months post-weaning phase. This post-weaning phase may be a critical period demanding high levels of allomaternal support because maternal caregiving decreases while toddlers are still heavily dependent on care. Moreover, toddlerhood is also the period characterized by increases in parent-child conflict related to the child's burgeoning autonomy and parental disciplinary strategies (55), thereby increasing caregiving load for parents. According to the grandmother hypothesis (22), the prolonged post-reproductive lifespan of grandmothers is the result of evolution favoring post-reproductive individuals their fitness through assisting their own offspring to reproduce successfully (49). Our results add to these findings and suggest that, under the adverse COVID-19 conditions, grandparents indirectly promote children's well-being by exerting protective effects on the rearing environment.

Grandparental involvement was, however, only an important predictor in the top winning model predicting maternal harshness in China, but not in the Netherlands and Italy. This is consistent with our previous study with the same sample in which we found that grandparental support only lowers mental health problems in Chinese mothers (31). Hence, no grandparent effect was observed in Italy and the Netherlands, possibly because in these countries the nuclear family is the most common family constellation, and nonresidential grandparents were kept at a distance from parents and grandchildren during the lockdown. Another remarkable difference between the Dutch and Italian vs. the Chinese models, potentially related to cultural variations in family structure, was that the number of children contributed to harsh care in the Netherlands and Italy, whereas this factor was considered unimportant in the Chinese model. Although previous research has identified a large number of children in the home as a risk factor for child maltreatment (48), these studies were predominately conducted in Western societies with nuclear families. In extended families, grandparents or other kin may assist with child care in the home environment, thus sharing the caregiving load and allowing parents to have more children without increasing the risk for child maltreatment (49). In China, where the extended family is considered traditional, a large number of children may therefore be a less important predictor for maltreatment. These results suggest that the antecedents of harsh parenting during the lockdown may be different across countries due to cultural variations in family composition. This interpretation is supported by our observation that Dutch risk factors predicted harsh care of Italian mothers reasonably well, possibly because in both countries the nuclear family is most prevalent, whereas Dutch and Italian models performed poorly in predicting harsh parenting in China. It should be noted that many countries are multicultural and include multiple ethnic groups. Hence, our findings do not only indicate that there is no universal risk factor model that can be used for the identification of at-risk families, but also warrant caution against accepting one model for COVID-19-related risk factors within one country. Cultural variations in family composition may accentuate or minimize the importance of risk and protective factors, possibly leading to between- and within country differences in the constellation of risk factor models.

In addition to the potential role of family composition, employment rates of mothers may also have resulted in a differential constellation of predictors across the three countries. The employment rate of the Chinese mothers sample was very high in the current sample (93.6% of mothers), which matches well with the above world-average record of female labor force participation in China (56). Moreover, the vast majority of women are involved in full-time employment as part-time working has not yet been initiated/stimulated in China (57). As a consequence, the need of allomaternal support may be high in China: Chinese mothers may need support with childcare from either grandparents or father in order to meet the demands from work (58). This may explain why Chinese mothers who benefitted from support from highly involved fathers showed lower levels of harsh parenting, whereas father involvement was not considered an important predictor in Italy and the Netherlands. In line with this explanation, we found that father involvement was higher in China compared to Italy and the Netherlands. Another unexpected finding was that work-related stress or work-related changes predicted harsh parenting in the Netherlands and China, but not in Italy. In Italy, the male breadwinner model is most prevalent and female employment rates are rather low (59). Although work-related changes and stress reported by Italian mothers was quite high and the majority of mothers were employed, her partner's financial and job security may have lowered maternal stress regarding financial resources and buffered the effect of mothers' work stress on parenting abilities.

During COVID-19, in particular older adults were advised to keep social distance and (non-residential) grandparents who were involved in child care prior to the pandemic suddenly refrained from babysitting. Although this may have been a necessary precaution in order to avoid exposure to the virus, loss of allomaternal support from grandparents may have had a negative impact on parents (31) as well as children. The unexpected loss of grandparental support during the lockdown may have increased parenting stress, which may in turn leads to an overreliance on less effective disciplinary strategies, such as harsh discipline. Although grandparental involvement in child care exerts positive influences on children's health and well-being (9), the role of grandparents in caregiving is still sidelined in policy decisions. Research on caregiving also focused mainly on the mother as the primary caregiver and neglected the role of other caregivers such as grandparents. Our finding that high levels of allomaternal support from grandparent and father reduces the risk for harsh maternal caregiving during the lockdown in China underscores the importance of shared care, and may inform policies regarding child care during future pandemics. Adopting approaches to build a pandemic-proof community of care and strengthening networks of support inside and outside the family unit may help at-risk parents during future pandemics.

Some strengths and limitations should be noted. One strength of the study is that we examined the cross-cultural validity of factors predicting harsh care using large samples from three different countries. Examining parenting during the pandemic across countries is important because COVID-19 is a global crisis and understanding factors predicting harsh care will help identifying at-risk families during future pandemics. Yet, it is unclear whether results from individual countries are replicable across countries. Another strength is the use of cross-validation, which enabled us to identify those predictors that best predict maternal harshness in our data, but also perform well in predicting harsh parenting in various random subsets of the data. Cross-validation therefore revealed models that can be used to predict harsh parenting during future pandemics. This contrasts with standard statistical analyses that risk overfitting their regression models, resulting in models that fit the initial data very well, but are difficult to replicate in future research.

Another strength is that allomaternal support from father was measured with a 20-item task division questionnaire, enabling us to study how degree of paternal involvement impacts on maternal caregiving. However, it should be noted that grandparental involvement was measured dichotomously and we were not able to differentiate between maternal and paternal grandparents. Effects of grandparental involvement may be even more pronounced with continuous measures with more power. A second limitation is that some variables did not have sufficient within-country variability to test whether they contributed to harsh care. For example, in the Netherlands almost all parents reported living in a house with a private garden. In contrast with our expectation that lower quality housing would predict harsh care, living in a house with a garden was related to higher levels of harsh parenting in Italy. This effect, however, only approached significance in the robust regression analysis, was absent in China, and may therefore be the result of confounding factors that we did not control for in the current study. In addition, it should be noted that the Chinese, Italian, and Dutch samples showed differences in sociodemographic variables, such as age and employment. However, due to the large sample size, statistical tests were sensitive to detect very small differences between countries. It is not very likely that this has influenced the results, as effect sizes were small and we controlled for sociodemographic variables in all analyses. The analyses also mainly focused on predictive models in which multivariate associations are more important than mean level differences between the countries. Furthermore, Italy was affected to a larger extent by COVID-19 than the Netherlands and China. During data collection, China was in the aftermath of COVID-19, whereas the number of infections were still high in Italy and the Netherlands. Pandemic restrictions concerning closures of schools and day care centers, social distancing, and remote working were, however, the same across countries. Moreover, our results show that COVID-19-related health concerns did not contribute to the prediction of harsh parenting. It is therefore unlikely that the constellation of factors predicting harsh care differed across countries due to differences in COVID-19 severity. Furthermore, it should be noted that the threshold parameters in the harsh parenting factor model for ordinal items were not invariant across countries, implying that factors other than harsh parenting were influencing the differences between countries on some harsh parenting item scores. The deviation from invariance however seemed small and invariance did hold for factor loadings. This analysis suggests that mean differences between countries on the harsh parenting scale should be interpreted with care. Lastly, we examined only maternal harshness and excluded fathers from the current analyses although we did examine paternal involvement in child care. Future COVID-19 studies should involve fathers. Moreover, future research should also examine the impact of lockdowns in families at risk for maltreatment. Allomaternal support may be particularly important in at-risk families. For example, a high-quality relationship with involved grandparents may play a buffering role for children in at-risk families.

In conclusion, during COVID-19 parents were presented with unprecedented challenges. For some families, pandemic-related distress may interferes with adequate parenting. Examining risk and protective factors for impaired parenting is therefore important and will help identifying at-risk families during COVID-19 and future pandemics. Our study showed that the constellation of factors predicting maternal harsh parenting during the COVID-19 lockdown is not identical across countries. Although marital conflict and maternal psychopathology are shared risk factors, the predictive performance of models predicting harsh parenting during COVID-19 differed across countries. Hence, the constellation of factors predicting maternal harshness during COVID-19 is not universal. This information will be valuable for the identification of at-risk families during future pandemics. Importantly, our results indicate that shared childrearing can buffer against risks for harsh parenting during adverse circumstances such as COVID-19, thus motivating the development of pandemic-proof support approaches, customized for individual countries, to assist parents with childcare and reduce parenting stress during future pandemics. During the lockdown, in the absence of any childcare support from community, the concept “It takes a village to raise a child” (8) may have had more meaning than ever. Mothers do not rear children on their own and allomaternal support from fathers, grandparents, and the community may be needed to establish resilience at a family level. Hence, building a pandemic-proof community of care can be leveraged in efforts to prevent harsh caregiving practices and their detrimental effects on children's well-being during future pandemics.

## Data Availability Statement

The raw data supporting the conclusions of this article will be made available by the authors, without undue reservation.

## Ethics Statement

The studies involving human participants were reviewed and approved by School of Social and Behavioral Sciences of Tilburg University, Department of Psychology of Padua University, Peking University Medical Ethics Board. The patients/participants provided their written informed consent to participate in this study.

## Author Contributions

MR: conceptualization, investigation, validation, data curation, writing—original draft, funding acquisition, supervision, project administration, and resources. PL: software, methodology, validation, data curation, formal analysis, visualization, and writing—original draft. MV-V: investigation, writing—review, and editing. MB-K and MvIJ: methodology, supervision, writing—review, and editing. PDC and JG: investigation, data curation, writing—review editing, resources, and funding acquisition. All authors contributed to the article and approved the submitted version.

## Funding

This work was supported by the National Social Science Fund of China (Number: 20VYJ042) to JG and a corona fast-track data grant from the Netherlands Organization for Scientific Research (NWO; 440.20.013) awarded to MR. MB-K was funded by the European Research Council (ERC AdG) and the Netherlands Organization for Scientific Research (NWO grant number 024.001.003).

## Conflict of Interest

The authors declare that the research was conducted in the absence of any commercial or financial relationships that could be construed as a potential conflict of interest.

## Publisher's Note

All claims expressed in this article are solely those of the authors and do not necessarily represent those of their affiliated organizations, or those of the publisher, the editors and the reviewers. Any product that may be evaluated in this article, or claim that may be made by its manufacturer, is not guaranteed or endorsed by the publisher.
