# Supplementary material for: Predictive Models of Maternal Harsh Parenting During COVID-19 in China, Italy, and Netherlands
Source: Front Psychiatry. 2021 Sep 8;12:722453. doi: 10.3389/fpsyt.2021.722453 (PMC8455908; doi:10.3389/fpsyt.2021.722453)
Supplement: Supplementary file 1 [file Data_Sheet_1.docx]

**Results**

Table S1. Information regarding quarantine and COVID-19 diagnoses.

| **COVID-19 quarantine** | IT  (N=641) | NL  (N=900) | CH  (N=922) | *p*-value | *Cramer V* |
| --- | --- | --- | --- | --- | --- |
| Forced quarantine due to  medically confirmed  infection | 30 (4.7%) | 20 (2.2%) | 9 (1.0%) | <.001 | .10 |
| Voluntary quarantine due to  confirmed infection | 50 (7.8%) | 67 (7.4%) | 17 (1.8%) | <.001 | .12 |
| Voluntary quarantine due to  fear of infection | 190 (29.6%) | 167 (18.6%) | 61 (6.6%) | <.001 | .24 |
| **COVID-19 diagnosis mother** | 4 (0.6%) | 3 (0.3%) | 1 (0.1%) | .211 | .04 |
| **COVID-19 diagnosis partner** | 2 (0.3%) | 3 (0.3%) | 0 (0%) | .222 | .04 |

Table S2: For each country, the predictors of harsh parenting according to the top 3 regression models identified through cross validation. * This indicates the percentage of the 200 cross validation repeats a particular model showed lowest RMSE of all 13311 investigated models.

| Predictors | Italy | | | Netherlands | | | China | | |
| --- | --- | --- | --- | --- | --- | --- | --- | --- | --- |
| Model ranking | 1 | 2 | 3 | 1 | 2 | 3 | 1 | 2 | 3 |
| Number of wins* | 46% | 34% | 6% | 40% | 19% | 11% | 47% | 29% | 6% |
| Number of children | x | x | x | x | x | x |  |  | x |
| Age youngest child |  |  |  |  |  |  |  |  |  |
| Education | x | x | x |  |  |  | x | x |  |
| Income |  |  | x |  | x |  | x |  | x |
| House with garden | x | x | x |  |  |  |  |  |  |
| Work changes mother |  |  |  | x | x | x |  |  |  |
| Work stress mother |  |  |  |  |  |  | x | x | x |
| COVID-19 health concerns |  |  |  |  |  |  |  |  |  |
| General psychopathology | x | x | x | x | x | x | x | x | x |
| Marital conflict | x | x | x | x | x | x | x | x | x |
| Father involvement |  | x |  |  |  |  | x | x | x |
| Grandparents childcare |  |  |  |  |  | x |  |  |  |
| Other childcare |  |  |  |  |  |  |  |  |  |
| Grandparents childcare *   Age youngest child |  |  |  |  |  |  | x | x | x |
| Grandparents childcare *   Father involvement |  |  |  |  |  |  |  |  |  |

Table S3. Pearson correlations between psychological aggression and physical assault, childcare involvement of fathers, work-related distress, depression, anxiety, and posttraumatic stress disorder in the Italian sample. * *p* < .05, *** *p* < .001

|  | 1 | 2 | 3 | 4 | 5 | 6 | 7 |
| --- | --- | --- | --- | --- | --- | --- | --- |
| 1. Work stress |  |  |  |  |  |  |  |
| 2. Anxiety | .27*** |  |  |  |  |  |  |
| 3. Depression | .26*** | .98*** |  |  |  |  |  |
| 4. Somatic symptoms | .26*** | .94*** | .93*** |  |  |  |  |
| 5. PTSD symptoms | .27*** | .99*** | .99*** | .94*** |  |  |  |
| 6. Father involvement | 0.03 | -.07 | .10* | -0.7 | -.08* |  |  |
| 7. Physical assault | .11*** | .22*** | .22*** | .19*** | .22*** | -0.02 |  |
| 8. Psychological aggression | .06 | .18* | .16*** | .17*** | .17*** | -.04 | .57*** |

Table S4. Pearson correlations between psychological aggression and physical assault, childcare involvement of fathers, work-related distress, depression, anxiety, and posttraumatic stress disorder in the Dutch sample. ** *p* < .01, *** *p* < .001

|  | 1 | 2 | 3 | 4 | 5 | 6 | 7 |
| --- | --- | --- | --- | --- | --- | --- | --- |
| 1. Work stress |  |  |  |  |  |  |  |
| 2. Anxiety | .21*** |  |  |  |  |  |  |
| 3. Depression | .21*** | .99*** |  |  |  |  |  |
| 4. Somatic symptoms | .18*** | .96*** | .94* |  |  |  |  |
| 5. PTSD symptoms | .21*** | .99*** | .99* | .96*** |  |  |  |
| 6. Father involvement | .05 | -.16*** | -.16** | -.16*** | -.16*** |  |  |
| 7. Physical assault | .13*** | .25*** | .27*** | .24*** | .26*** | -.08** |  |
| 8. Psychological aggression | .13*** | .15*** | .16*** | .15*** | .15*** | -.01 | .66*** |

Table S5. Pearson correlations between psychological aggression and physical assault, childcare involvement of fathers, work-related distress, depression, anxiety, and posttraumatic stress disorder in the Chinese sample. ** *p* < .01, *** *p* < .001

|  | 1 | 2 | 3 | 4 | 5 | 6 | 7 |
| --- | --- | --- | --- | --- | --- | --- | --- |
| 1. Work stress |  |  |  |  |  |  |  |
| 2. Anxiety | .11*** |  |  |  |  |  |  |
| 3. Depression | .11*** | .99*** |  |  |  |  |  |
| 4. Somatic symptoms | .09** | .98*** | .97*** |  |  |  |  |
| 5. PTSD symptoms | .11** | .99*** | .99*** | .98*** |  |  |  |
| 6. Father involvement | -.07* | -.08*** | -.09** | -.08** | -.08** |  |  |
| 7. Physical assault | .11*** | .31*** | .31*** | .31*** | .31*** | -.15*** |  |
| 8. Psychological aggression | .11*** | .26*** | .26*** | .26*** | .26*** | -.14*** | .70*** |

1. de Rooij M, Weeda W. Cross-Validation: A Method Every Psychologist Should Know. *Advances in Methods and Practices in Psychological Science* (2020) 3(2):248-63. doi: 10.1177/2515245919898466.
